# Kupffer Cells and Blood Monocytes Orchestrate the Clearance of Iron–Carbohydrate Nanoparticles from Serum

**DOI:** 10.3390/ijms23052666

**Published:** 2022-02-28

**Authors:** Tasneem Arsiwala, Anne-Cathrine S. Vogt, Amy E. Barton, Vania Manolova, Felix Funk, Beat Flühmann, Martin F. Bachmann

**Affiliations:** 1 Department of Rheumatology, Immunology and Allergology, Inselspital, University Hospital Bern, 3010 Bern, Switzerland; tasneem.arsiwala@gmail.com (T.A.); martin.bachmann@dbmr.unibe.ch (M.F.B.); 2 Department for BioMedical Research (DBMR), University of Bern, 3010 Bern, Switzerland; 3 Vifor (International) AG, 9001 St. Gallen, Switzerland; amy.barton@viforpharma.com (A.E.B.); vania.manolova@viforpharma.com (V.M.); felix.funk@viforpharma.com (F.F.); beat.fluehmann@viforpharma.com (B.F.); 4 Nuffield Department of Medicine, Jenner Institute, University of Oxford, Oxford OX3 7BN, UK

**Keywords:** iron nanomedine, ferric carboxymaltose, monocytes, Kupffer cells, biodisposition

## Abstract

Intravenous (IV) iron nanoparticle preparations are widely used to treat iron deficiency. The mechanism of mononuclear phagocyte system-mediated clearance of IV iron nanoparticles is unknown. The early uptake and homeostasis of iron after injection of ferric carboxymaltose (FCM) in mice was studied. An increase in serum iron was observed at 2.5 h followed by a return to baseline by 24 h. An increase in circulating monocytes was observed, particularly Ly6C^hi^ and Ly6C^low^. FCM was also associated with a time-dependent decrease in liver Kupffer cells (KCs) and increase in liver monocytes. The increase in liver monocytes suggests an influx of iron-rich blood monocytes, while some KCs underwent apoptosis. Adoptive transfer experiments demonstrated that following liver infiltration, blood monocytes differentiated to KCs. KCs were also critical for IV iron uptake and biodegradation. Indeed, anti-Colony Stimulating Factor 1 Receptor (CSF1R)-mediated depletion of KCs resulted in elevated serum iron levels and impaired iron uptake by the liver. Gene expression profiling indicated that C-C chemokine receptor type 5 (CCR5) might be involved in monocyte recruitment to the liver, confirmed by pharmaceutical inhibition of CCR5. Liver KCs play a pivotal role in the clearance and storage of IV iron and KCs appear to be supported by the expanded blood monocyte population.

## 1. Introduction

Iron is an essential transition metal for metabolic homeostasis, hemoglobin synthesis as well as cell proliferation and survival [1]. Iron deficiency is a common condition worldwide and can result in severe clinical manifestations that include fatigue, malnutrition and cardiac hypertrophy [2]. The most common etiologies are insufficient iron absorption, blood loss, poor nutrition, and other coexisting comorbidities (e.g., kidney disease, heart failure, pregnancy/post-partum losses). 

Iron deficiency can be treated by iron supplementation via the oral or parenteral routes [3]. Oral iron supplements are available as ferrous and ferric salts. However, oral agents are generally poorly absorbed, especially in disease states with concomitant inflammation, and have adverse gastrointestinal effects that lead to poor patient adherence [4]. The preparations developed for intravenous (IV) administration are colloidal suspensions of iron (ferric oxyhydroxide)–carbohydrate nanoparticles (NPs) with particle sizes extending from 5 to 40 nm [5,6]. These iron–carbohydrate nanoparticle preparations have been shown to be more efficacious than oral iron supplements in disease states such as chronic kidney disease [7,8]. Second-generation IV iron nanomedicines have greater stability of the iron–carbohydrate complex and can be administered in larger doses [6]. The pathway and mechanisms by which IV iron–carbohydrate nanoparticle preparations are taken up and biodegraded by the mononuclear phagocytic system (MPS) has not been elucidated [9].

MPS cells are principally involved with the uptake of iron–carbohydrate nanoparticle preparations [10]. Tissue-resident macrophages, such as Kupffer cells (KCs) in the liver, play a pivotal role in iron metabolism [11]. While circulating blood monocytes are presumed to take up iron nanoparticles after IV injection, their exact role in iron metabolism and organ distribution has not been studied [12]. The objective of this exploratory study was to evaluate the kinetics of iron clearance and redistribution of iron to the liver after administration of ferric carboxymaltose (FCM) in mice.

## 2. Results

### 2.1. Clearance of Circulating Iron Carboxymaltose in the Serum Occured within 24 h

**Figure 1:**
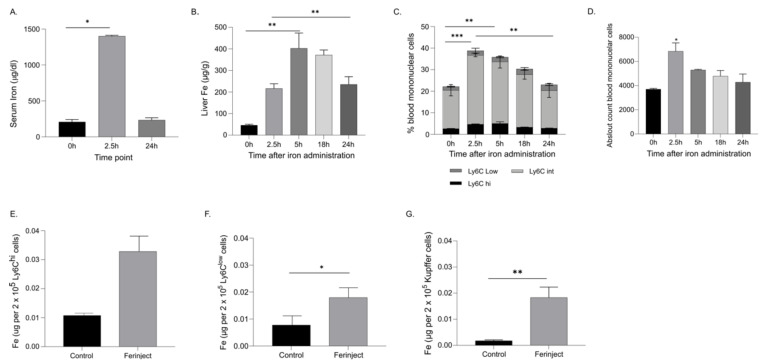
Iron is elevated in the serum, whole liver and blood monocytes after IV iron administration. (**A**) Serum iron levels measured at 2.5 h and 24 h after IV iron administration (**B**) Iron concentration measured in whole liver by ICP-MS. (**C,D**) Iron administration increased the percentage and absolute amount of circulating blood monocytes in a time-dependent manner; * denotes statistical significance in total monocytes. (**E,F**) Iron levels in Ly6C^hi^ and Ly6C^low^ blood monocytes at 2.5 h post IV iron administration. (**G**) Iron levels in liver KCs 2.5 h post IV iron administration. All measurements were taken using at least *n* = 3 individual mice, except for blood monocytes where the cells were pooled from 5 mice, and two independent measurements were taken. The data are expressed as Mean ± SEM. For (**A**–**D**), statistics performed by ordinary one-way ANOVA using Tukey’s multiple comparison test where the mean of each group is compared to the control group (0 h). * *p* < 0.05, ** *p* < 0.01, *** *p* < 0.001. For (**E**–**G**) Statistics performed by unpaired *t*-test, where the Ferinject group is compared to the control group. * *p* < 0.05, ** *p* < 0.01.

The concentration of iron was measured in the serum, blood monocytes, and liver tissue from mice at an early time point of 2.5 h and a later time point of 24 h post-administration to determine the distribution of iron after FCM administration (Figure 1). At the earliest timepoint assessed (2.5 h), a significant elevation of serum iron was observed, followed by a decrease to baseline concentration at 24 h (Figure 1A). Since the liver is a primary organ for iron storage, liver tissue iron concentrations were measured by ICP-MS. Peak liver tissue concentrations occurred at 5 h post-FCM administration (Figure 1B). Liver iron concentrations remained elevated at 24 h after iron administration compared to the baseline. The percentage of blood monocyte subsets, analysed by flow cytometry, and their iron content increased at 2.5 h after injection of FCM, particularly the Ly6C^hi^ and Ly6C^low^ subsets. Over time, the blood monocyte population declined, reaching baseline numbers at 24 h (Figure 1C,D). Iron concentration assessed by ICP-MS increased in Ly6C^hi^ and Ly6C^low^ monocyte populations at 2.5 h after FCM administration. (Figure 1E,F). Additionally, iron concentration increased in isolated KCs at 2.5 h post iron administration (Figure 1G). 

### 2.2. FCM Administration Increased Blood and Liver Monocytes and Simultaneously Decreased Liver Kupffer Cells

**Figure 2:**
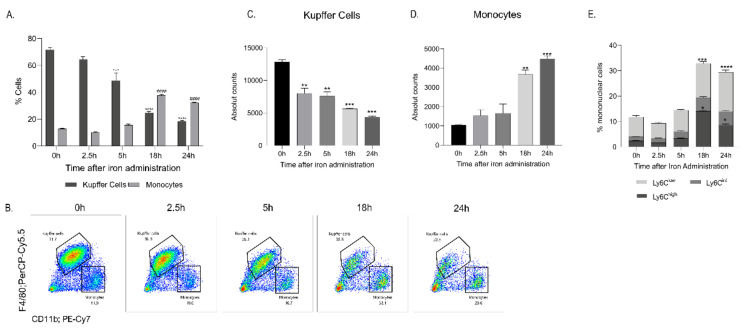
FCM administration increased liver monocytes and decreased liver Kupffer cells**.** Livers were collected at different timepoints 0 h, 2.5 h, 5 h, 18 h and 24 h after IV FCM administration and liver monocytes and KC populations were analysed by flow cytometry. (**A**–**D**) Flow cytometry analysis of 1 × 10^6^ liver cells revealed that upon IV FCM administration, a decrease in liver Kupffer cells (KC) and an increase in liver monocytes are observed. (**E**) Subset analysis showed the increased liver monocytes are of the Ly6C^hi^ and Ly6C^low^ subsets. Gating done on live, single, CD45+, Ly6G-, CD3- cells. * denotes statistical significance in KC percentage; # denotes statistical significance in monocyte percentage. All measurements were taken at least twice on *n* = 3. The data are presented as Mean ± SEM. Statistics were performed by ordinary one-way ANOVA using Tukey’s multiple comparison test where the mean of each group is compared to the control group (0 h). * *p* < 0.05, ** *p* < 0.01, *** *p* < 0.001, ****^/^#### *p* < 0.0001.

As iron concentration was shown to increase in the liver and liver KCs upon FCM administration in mice, liver monocytes and KCs were characterized over 24 h by flow cytometry to determine whether circulating monocytes are recruited to the liver. Flow cytometric analyses revealed a time-dependent increase in the percentage of liver monocytes at time points 2.5 h, 5 h, and 18 h, paralleled by a co-temporal decline in the percentage of KCs (Figure 2A–D). Further analyses of the liver monocytes revealed that the monocytes are of the Ly6C^hi^ and Ly6C^low^phenotype (Figure 2E). These findings suggest that the increase in liver monocytes may result from the recruitment of iron-rich circulating blood monocytes to the liver to clear circulating FCM nanoparticles. Although the exact mechanism of liver KC decline was not investigated, a time-dependent transient increase in annexin V, an apoptosis marker, was observed in the KCs, when analysed with flow cytometry. (Supplementary Figure S1). 

### 2.3. Adoptive Transfer Demonstrated Recruitment of Blood Monocytes to the Liver

**Figure 3:**
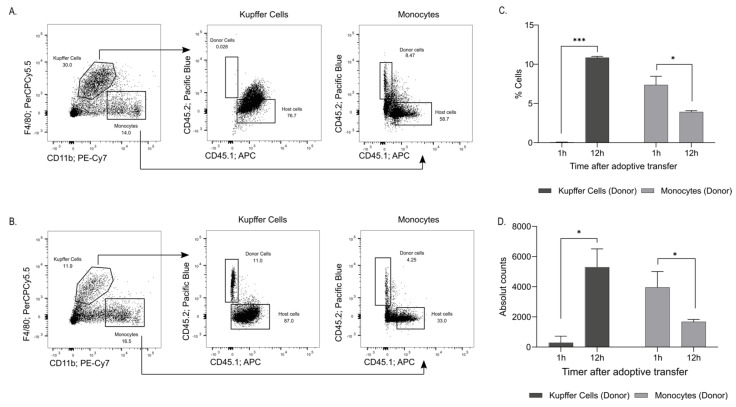
Adoptive transfer of CD45.2 donor monocytes tracks their recruitment to the liver of CD45.1 recipients**.** FCM was administered IV into CD45.2 mice (donor). After 2.5 h, the blood was collected from mice, and the monocytes were purified and injected IV into CD45.1 mice (recipient). 1 h and 12 h post-monocyte transfer, the livers were analysed to determine the percentage of donor-infiltrated cells. (**A**) 1 h post-monocyte transfer, about 8.5% of the monocytes and no KCs were from the circulating donor cells, (**B**) 12 h post-monocytes transfer, 4.25% of the liver monocytes were iron-rich donor CD45.2 cells, while 11% of the KCs were derived from the donor cells. These are representative data from two independent experiments. (**C**,**D**) Bar graph representation of KC and monocytes from donor mice (CD45.2) found in the recipient liver (CD45.1 mice). For the transfer of monocytes from the CD45.2 into CD45.1, blood cells were pooled from 7 mice. For (**C,D**) statistics performed by unpaired *t*-test, where the 12 h group is compared to the 1 h group. * *p* < 0.05, *** *p* < 0.001.

Monocytes circulating in the blood have long been considered to act as precursors of tissue-resident macrophages [13]. To demonstrate that the increase in liver monocytes was a result of circulating monocytes, an adoptive transfer was performed. FCM 40 mg/kg was administered (IV) to CD45.2 donor mice. At 2.5 h post-administration, blood monocytes were isolated and transferred to CD45.1 recipient mice to follow cellular tracking. Flow cytometric analyses of the liver cells performed 1 h post-monocyte transplantation revealed that approximately 8.5% of the liver monocytes already comprised the newly recruited donor CD45.2 cells, whereas essentially all KCs were from the recipient mice at this early time point (Figure 3A). Analyses at 12 h post-transfer revealed that approximately 4.25% of the liver monocytes were donor CD45.2 cells, while 11% of the KCs were now derived from the donor cells (Figure 3B–D). This experiment demonstrated that iron-rich blood monocytes are recruited to the liver and that the newly recruited liver monocytes differentiate into KCs.

### 2.4. Anti-CSF1R Antibody Administration Depleted Tissue-Resident Macrophages Disrupting Iron Uptake and Metabolization

**Figure 4:**
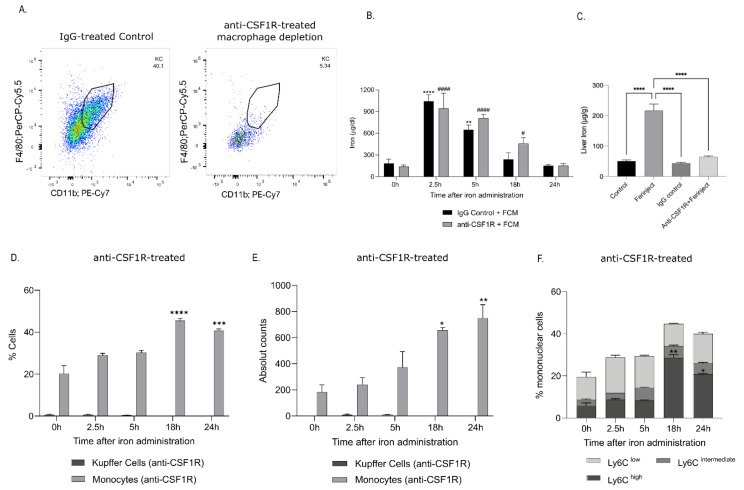
Macrophage depletion increased serum iron concentrations and reduced the amount of iron delivered to the liver. Macrophage depletion was induced by injecting an anti-CSF1R antibody. (**A**) Representative dot plots demonstrating that anti-CSF1R treatment effectively depleted liver KCs. Gating was performed on live single CD45+ Ly6G- cells. In mice with macrophage depletion, we measured (**B**) serum iron levels 0 h, 2.5 h, 5 h, 18 h and 24 h post FCM administration, *denotes statistical difference in serum iron of IgG control mice, # denotes statistical difference in serum iron in anti-CSF1R treated mice (**C**) whole liver iron levels 2.5 h post FCM injection, and (**D**,**E**) infiltration of blood monocytes in the liver, independently from KC depletion. (**F**) Percentage of monocyte subsets in the liver of KC-depleted mice 0 h, 2.5 h, 5 h, 18 h and 24 h post FCM administration. The number of mice was at least *n* = 4 in two independent experiments. The data are expressed as Mean ± SEM. Statistical significance was calculated using ANOVA, followed by Bonferroni’s multiple comparison test as a post hoc test, where for (**C**), the mean of each group is compared to every other group and for (**B**,**D**–**F**) the mean of each group is compared to the control group (0 h). *^/#^ *p* < 0.05, ** *p* < 0.01, *** *p* < 0.001, ****^/####^ *p* < 0.0001.

In mice with depleted liver KCs (Figure 4A), serum iron was not cleared as efficiently compared to control IgG-treated mice, and serum iron was elevated 18 h post FCM administration (Figure 4B). Additionally, the iron content of the liver was reduced at 2.5 h after FCM injection (Figure 4C). However, KC depletion in the liver did not prevent the recruitment of monocytes to the liver after FCM injection, as they were present in the liver at similar percentages as in control mice (Figure 4D,E). Similar to control mice, the infiltrating monocytes were of the Ly6C^hi^ subtype in KC-depleted mice (Figure 4F). These data show that depletion of tissue-resident KCs resulted in elevated serum iron levels and impaired iron uptake by the liver.

### 2.5. Recruitment of Blood Monocytes into the Liver Was CCR5-Mediated

**Figure 5:**
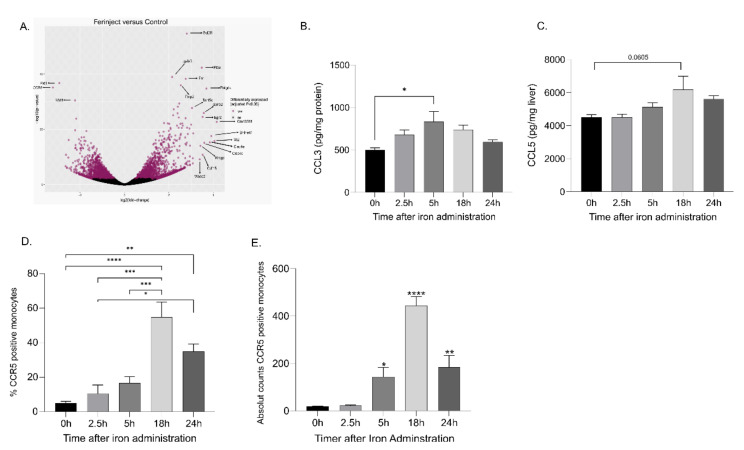
CCR5 played a role in the recruitment of blood monocytes into the liver. RNA-seq analysis showed that the CCR5 gene is downregulated in KCs isolated from mice injected with FCM. (**A**) Volcano plot depicts −log10 Benjamini–Hochberg-corrected *p*-value versus log2 fold regulation for each gene for KCs sorted from FCM and saline (control)-treated mice after 2.5 h, (**B**) CCL3 levels increase and (**C**) CCL5 levels show a trend of increasing 5 h post-IV iron administration in liver lysate. (**D**,**E**) CCR5 expression on circulating blood monocytes post-FCM treatment by flow cytometry. The number of mice is *n* = 3 for RNA-seq experiments and two independent experiments of *n* = 5 for chemokine measurements. The data are expressed as Mean ± SEM. Statistical significance was calculated using ANOVA, followed by Bonferroni’s multiple comparison test as a post hoc test. where the mean of each group is compared to the control group (0 h). * *p* < 0.05, ** *p* < 0.01, *** *p* < 0.001, **** *p* < 0.0001.

RNA-seq analyses on KCs isolated from control and FCM-treated mice showed that CCR5 mRNA expression was significantly downregulated in FCM-treated mice (Figure 5A). These data were confirmed at the protein level by flow cytometry analysis of KCs (Supplementary Figure S2). Similarly, C-C motif chemokine ligand 3 (CCL3), (one of the ligands of CCR5) levels in liver lysates increased over time following FCM administration, with maximal concentrations observed at 5 h. Additionally, we found C-C motif chemokine ligand CCL5 (ligand of CCR5) concentrations increased, with the highest concentrations observed at 18 h, although this was not statistically significant (*p* = 0.0605) (Figure 5B,C). The percentage of CCR5-positive cells was also significantly elevated from baseline in circulating blood monocytes in mice receiving FCM at 18 h and 24 h post injection. (Figure 5D,E).

### 2.6. Blocking CCR5 Inhibited the Migration of Blood Monocytes to the Liver

**Figure 6:**
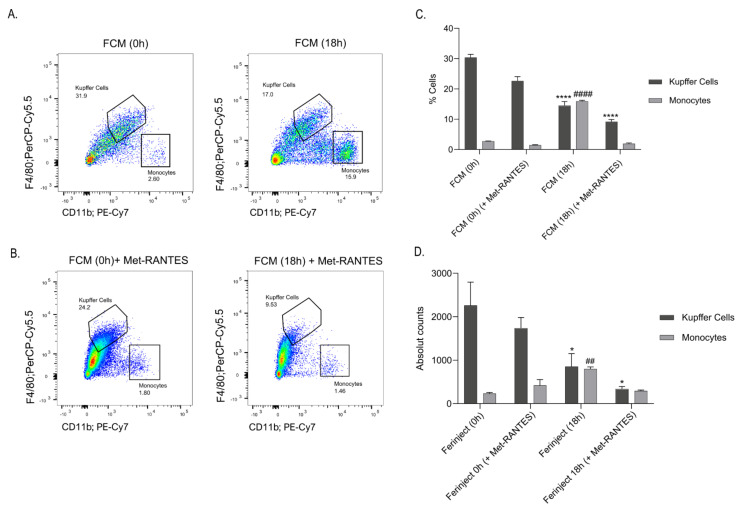
Met-RANTES reduced infiltration of blood monocytes into the liver. (**A**–**D**) Treatment of 5 ug Met-RANTES reduced the infiltration of blood monocytes into the liver 18 h post-iron treatment. The number of mice was *n* = 3 in two independent experiments. * denotes statistical significance in KC percentage and absolute amounts; # denotes statistical significance in monocyte percentage and absolute amounts. The data are expressed as Mean ± SEM. Statistics performed by ordinary one-way ANOVA using Tukey’s multiple comparison test where the mean of each group is compared to the control group (0 h), followed by Bonferroni’s multiple comparison test as a post hoc test. * *p* < 0.05, ^##^ *p* < 0.01, ****/#### *p* < 0.0001.

As an increase in CCR5-expressing monocytes in the liver of NP treated mice was observable, we next investigated whether the recruitment of monocytes to the liver relies on CCR5-mediated chemotaxis. To this end, we blocked CCR5 using Met-RANTES, a partial CCR5 antagonist (Figure 6A,B). At 18 h post FCM administration, there was no monocyte infiltration in the liver of mice treated with the competitive CCL5 inhibitor, Met-RANTES, and the percentage of infiltrating monocytes was similar to those observed in saline-treated control mice (Figure 6C,D).

## 3. Discussion

Oral iron preparations often fail to replete iron stores adequately due to limited absorption and poor tolerability [14]. In contrast, IV iron–carbohydrate nanomedicines circumvent oral absorption issues, and these agents (e.g., FCM) can be given at higher single doses. Therefore, IV iron–carbohydrate preparations are widely used to treat iron deficiency. There are different iron–carbohydrate nanoparticle preparations available on the market and they differ in their physicochemical characteristics [6]. The surface characteristics of the complex, in part, dictate the rate and extent of macrophage uptake and biodegradation and differ among the commercially available preparations [11,15]. Serum ferritin is a pharmacodynamic marker of the incorporation of iron derived from iron–carbohydrate complexes into the physiological iron stores. A recent study in human subjects showed differential area-under-the plasma concentration time curve profiles for serum ferritin after administration of three IV iron–carbohydrate preparations (iron sucrose, iron isomaltoside 1000 and FCM) [16]. In this study, we provide novel experimental evidence that elucidates a mechanism of clearance of iron from the serum after IV iron–carbohydrate nanoparticle administration and reveals potential synergy of circulating blood monocytes and liver KCs to efficiently deliver iron to the liver. 

We observed that serum iron concentrations increased significantly at 2.5 h after FCM administration. However, 24 h later, serum iron levels returned to baseline. It should be noted that serum iron measured by QuantiChrome detects transferrin-bound iron as well as a small amount of the iron released from the NPs. Iron delivery to the liver appears to be slower with an increase in the liver iron concentrations observed at 5 h after FCM injection. Liver iron concentrations remained above the levels measured in control animals at 24 h, indicating that iron is retained for storage before being released for further utilization. Blood monocytes are among the first cells to encounter iron–carbohydrate nanoparticles after IV administration. Indeed, this study showed that FCM nanoparticles are taken up by monocytes in the bloodstream and this is followed by increased liver iron content, presumably due to subsequent transport and deposition in the liver. These data are consistent with the hypothesis that the liver is the primary organ of iron storage and crucial to iron–carbohydrate nanomedicine biodistribution. 

Interestingly, we observed an early and significant increase in iron concentrations in blood monocytes, confirming the importance of peripheral blood monocytes in early FCM clearance and distribution. At 2.5 h, following FCM administration, the percentage of blood monocytes increased for all three subsets (Ly6C^hi^ Ly6C^int^ Ly6C^low^). Thus, this suggests a coordinated response to facilitate the FCM clearance from the bloodstream. The Ly6C^hi^ and Ly6C^low^ monocytes are the classical and non-classical monocytes and have been shown to infiltrate the liver in other conditions to resolve tissue inflammation [17,18,19]. The percentage of blood monocytes returned to baseline by 24 h. Iron homeostatic sensors (intracellular and extracellular) and/or cytokine activation may contribute to upregulated peripheral blood monocyte production. After IV iron–carbohydrate nanomedicine administration, these iron-rich blood monocytes may migrate to the liver to deliver iron for further storage, biodegradation and/or transport [20]. 

A time-dependent decrease in KCs was observed with a nadir at 18 h. This decrease in KCs may have been compensated for by newly recruited iron-rich peripheral blood monocytes. These monocytes that appeared to be recruited to the liver may have subsequently differentiated into KCs and could explain the higher percentage of KCs shown in the liver at 24 h. The mechanism of reduction in the number of KCs after FCM administration is not entirely clear. KCs express genes involved in uptake and storage of iron [21] but the mechanisms by which iron–carbohydrate nanoparticles are endocytosed have not been fully elucidated [22,23]. Nevertheless, it has been shown that superparamagnetic iron–carbohydrate nanoparticles are mainly taken up by phagocytosis and degraded in the lysosome [24,25,26].

The increased percentage of annexin V^+^ KCs after FCM administration suggests that iron-rich KCs may undergo apoptosis [27]. However, whether this is iron mediated (e.g., ferroptosis) was not determined in this study [27]. Alternatively, and not mutually exclusively, the iron-rich KCs may leave the liver, a possibility supported by the observation that KCs rapidly down-regulate CCR5 upon FCM treatment, the chemokine receptor that recruits iron loaded monocytes to the liver before they differentiate into a new wave of KCs [28].

The adoptive transfer experiments revealed that the increase in liver monocytes results from infiltration by the peripheral blood monocytes. It is possible that these blood monocytes may eventually differentiate in the liver to KCs and repopulate the diminished KC pool; however, testing this assumption requires further studies. During embryonic development, the KCs are replenished by foetal liver monocytic precursors [19,29,30]. However, it has been found that under conditions associated with marked Kupffer cell depletion, bone-marrow-derived monocytes are recruited to the liver where they then mature into KCs [31]. It has also been demonstrated that Ly6C^high^ monocytes act as progenitors of the repopulated KCs [31].

However, these studies have not determined whether the circulating monocytes are able to differentiate in tissue-resident macrophages involved in iron uptake and storage following IV iron administration [32]. To test this, the depletion of issue-resident macrophages was induced by targeting the CSF1R. The anti-CSF1R antibody did not affect circulating blood monocytes, and these blood monocytes were still able to infiltrate the liver. Interestingly, although FCM administration in these macrophage-depleted mice further increased serum iron levels, liver iron concentrations decreased, as compared to the control mice. This demonstrates that both blood monocytes and tissue-resident macrophages are fundamental in the uptake, storage and biodegradation of iron–carbohydrate complexes.

The recruitment of cells into organs is mediated by chemokine-induced chemotaxis. RNA-sequencing of KCs isolated from FCM-treated mice at 2.5 h revealed a decrease in the CCR5 gene expression in the FCM treated group compared to control. Increased production of the CCR5 ligands CCL3 and CCL5 in the liver tissue was also observed. Analysis of CCR5 expression on blood monocytes revealed a time-dependent increase in CCR5 expression after FCM administration. Monocytes expressing CCR5 receptor can migrate along a chemotactic gradient binding the chemokine ligands CCL3, CCL4 and CCL5 [33]. Blocking CCR5 reduced the recruitment of blood monocytes into the liver at 18 h. These findings suggest monocyte infiltration in the liver could be CCR5 dependent.

Threurl et al. showed an on-demand mechanism to clear stressed erythrocytes with cooperation from Ly6C^hi^ blood monocytes which enter the liver and differentiate into transient liver macrophages first and then into iron-recycling KC-like cells [34]. Although a transient macrophage-like population was not identified in this study, we observe a similar increase in blood Ly6C^hi^ monocytes which differentiate into KCs in the liver and confirm the liver as a primary organ supporting iron–carbohydrate nanoparticle clearance from plasma after FCM administration.

The results of this work should be considered in the context of the following limitations. This series of experiments were focused on only one iron–carbohydrate nanomedicine and due to the heterogeneity in surface characteristics and other physicochemical properties, other iron–carbohydrate nanomedicines would likely exhibit different kinetic profiles for uptake and biodistribution [5,11,24]. Additionally, no studies were conducted to evaluate reactive species generation, in particular lipid peroxides, in either monocytes or KCs. Thus, the association of the annexin 5 positivity with ferroptosis cannot be concluded [35]. Finally, it is well established that nanoparticle interaction with plasma proteins results in adsorption and formation of hard and soft protein coronas, which contribute to the uptake profile, and thus our model may not be fully indicative of clearance and biodisposition kinetics in humans [36].

In summary, we show Ly-6C^high^ and Ly6C^low^ blood monocytes and KCs are the primary myeloid cells for iron–carbohydrate nanoparticle clearance after FCM administration. The circulating monocyte population expands rapidly and phagocytizes the iron–carbohydrate complex before being recruited to the liver in a CCR5-dependent manner. KCs also phagocytize iron, downregulate CCR5 and likely a small portion of the population undergoes apoptosis via a yet-to-be-determined mechanism. The recruited peripheral blood monocytes may differentiate to KCs to support the storage of FCM nanoparticles. Thus, FCM uptake by the blood monocytes and KCs largely contributes to the clearance of iron–carbohydrate nanoparticles within 24 h.

## 4. Materials and Methods

### 4.1. Animal Experiments

Experiments were performed in 8–10-week-old female C57Bl/6JRccHsd mice (Envigo, Amsterdam, The Netherlands). Mice received 40 mg elemental iron/kg of ferric carboxymaltose (Ferinject^®^, Vifor (International) AG, St. Gallen, Switzerland) diluted in saline by tail-vein injection. Serum iron was determined before IV iron 0 h and 2.5 h and 24 h post-iron administration. Liver iron concentrations as well as liver cell populations were analysed before IV iron 0 h and 2.5 h, 5 h, 18 h and 24 h post-iron administration. This timeline was used for the experiments throughout. Animals were sacrificed by CO_2_ inhalation per institutional protocol. The liver was then perfused by Hank’s Balanced Salt Solution (HBSS) and supplemented with 10 mM HEPES via the portal vein, and subsequent analyses and measurements were conducted. For macrophage depletion studies, mice were injected with 2 mg of anti-CSF1R antibody (BE0213, Bio X Cell, Lebanon, NH, USA) intraperitoneally as described previously [37,38] on day 0 and with 0.5 mg of anti-CSF1R antibody on day 7. Control was an IgG2a isotype control antibody (BE0085, Bio X Cell Lebanon, NH, USA). Liver tissue and blood samples were collected on day 10 for analyses. For the adoptive transfer experiments, 40 mg/kg of FCM was injected into CD45.2 mice (donor) by IV tail vein injection. After 2.5 h, blood was collected by cardiac puncture in coated (0.5 mM) EDTA tubes. Erythrocytes were lysed twice (#555899, BD Biosciences, Franklin Lakes, NJ, USA). Blood monocytes were purified (#19861, Stem Cell, Vancouver, CA, USA, Mouse Monocyte Isolation Kit) and transferred by tail vein injection into recipient CD45.1 mice. The number of cells transferred into control mice was 1.4 × 10^7^ cells/250 μl and for FCM treated mice 1.2 × 10^7^ cells/250 μL. After 1 h and 12 h, the livers of the recipient CD45.1 were removed. The recipient CD45.1 mice liver cells were assessed using flow cytometry to determine if there was any infiltration of CD45.2 donor cells present in the liver of recipient mice. To evaluate the effect of CCR5 blockade, mice were injected with 5 µg of Met-RANTES (#335-RM/CF, R&D Systems, Minneapolis, MN, USA) in 100 µL of sterile 0.9% saline once daily intraperitoneally on three consecutive days. On the third day of treatment, mice received 40 mg/kg of FCM diluted in sterile 0.9% saline by tail vein injection. At 18 h post-iron administration, liver tissue and blood samples were collected according to the previously described protocol. Isolated cells were then analysed by flow cytometry (BD Biosciences, BD^TM^ LSR II System, Franklin Lakes, NJ, USA).

### 4.2. Iron Measurements

Serum iron concentrates were measured by the QuantiChrom™ Iron Assay Kit (DIFE-250, BioAssay Systems, Hayward, CA, USA). Absorbance was measured using the SpectraMax M5 ELISA reader (Molecular Devices, San José, CA, USA) at a wavelength of 590 nm. Cellular (blood and liver monocytes, Kupffer cells) and liver tissue iron concentrations were measured by inductively coupled plasma mass spectrometry (ICP-MS) (NexIon 350, Perkin Elmer, MA, USA). For the liver tissue iron analysis, 100 mg of fresh tissue was sonicated in 100 µL 1× PBS. Prior to ICP-MS analysis, monocytes were sorted (see section cell sorting). For ICP-MS, samples were submitted to acidic digestion with 1 mL of concentrated HNO3 (69%, ROTIPURAN Supra, Roth) in quartz digestion vials using a microwave oven (synthWave, MWS Mikrowelle GmbH). The digestion protocol was the following: 5 min to heat up to 150 °C, 5 min to heat up to 180 °C and keep at 180 °C for 20 min. After digestion, the samples were diluted 50–100 times with 2% HNO_3_ solution, and iron content based on Fe56 was analysed by ICP-MS using KED mode with He as a collision gas on NexIon 350 D ICP-MS instrument (PerkinElmer, Waltham, MA, USA). Y was added as an internal standard at a concentration of 2 ppb to all the solutions and iron quantitation was performed using an external calibration curve with standards in the 0.05–50 ppb range. All measurements were performed in triplicate.

### 4.3. Collection of Liver Kupffer Cells

Kupffer cells and monocytes were isolated by adapting the protocol described by Xu et al. [39]. Briefly, liver Kupffer cells were obtained by initial vascular perfusion of the liver with 0.5 mM EDTA-HBSS solution through the portal vein. A single liver cell suspension was obtained by further incubating the liver with collagenase H (0.01%) and DNAse (0.01%) in DMEM containing 1 mM CaCl_2_, at 37 °C for 30 min. Following incubation, 5 mM EDTA solution (DMEM high glucose containing 5 mg/mL BSA) was added to liver suspensions to cease collagenase digestion by chelating Ca^2+^ ions. The liver homogenate was then filtered through a 100 µM cell strainer, and the suspension was centrifuged twice at 54× *g* for 3 min at 4 °C. Supernatants were then further centrifuged at 500× *g* for 5 min at 4 °C and then at 650× *g* for 5 min at 4 °C. The pellets were pooled in 25% Percoll and then loaded on 50% Percoll, both prepared from a 100% Stock Percoll (#170891012, Cytiva life sciences, Marlborough, MA, USA). The 25/50% loaded gradient was centrifuged at 4 °C for 30 min at 900× *g* (acceleration/deceleration at minimum speed). The non-parenchymal cells (NPCs) were collected from the interface of the two density layers. The collected NPCs were resuspended in FACS buffer (1xPBS supplemented with 2% FBS (#16250086, Thermo Fisher, Waltham, MA, USA) and centrifuged at 400× *g* for 5 min at 4 °C.

### 4.4. Collection of Blood and Blood Cells

The blood samples collected were transferred into coated (0.5 mM EDTA) tubes. Erythrocytes were lysed in RBC lysis buffer (#555899, BD Biosciences, Franklin Lakes, NJ, USA) and incubated on ice for 3 min. The process was stopped by adding FACS buffer, and the cell suspension was centrifuged at 400× *g* for 5 min. The lysis step was then repeated. 

### 4.5. RNA Extraction and Sequencing

Total RNA was extracted using the NucleoSpin^®^ RNA isolation kit (#740955, Macherey-Nagel Hoerdt, France) following the manufacturer’s instructions. The RNA integrity was verified using an Agilent Bioanalyzer 2100 (Agilent Technologies, Santa Clara, CA, USA) with an RNA Integrity Number of >8. The extracted total RNA was stored at −80 °C until sequencing.

High-quality RNA from all samples was processed for the preparation of cDNA libraries using an Illumina TruSeq Stranded mRNA Kit following the Illumina’s protocols. After quality control and quantification, cDNA libraries were pooled in groups of 6 technical replicates and sequenced on 5 lanes on the HiSeqTM 2000 (Illumina^®^ NEB, San Diego, CA, USA) to obtain approximatively 30 million reads (100 bp paired end) for each sample with insert sizes ranging from 200 to 400 base pairs.

### 4.6. ELISA

CCL3 and CCL5 were measured in liver lysates using the mouse CCL5/RANTES DuoSet ELISA (DY478-05, R&D systems, Minneapolis, MN, USA) and the mouse CCL3/MIP-1 alpha DuoSet ELISA (DY450-05, R&D systems, Minneapolis, MN, USA). Absorbance was measured at a wavelength of 450 nm using the SpectraMax M5 ELISA reader (Molecular Devices, San José, CA, USA).

### 4.7. Inhibition of Monocyte Recruitment by Met-Rantes

To determine whether the recruitment of monocytes into the liver is CCR5-receptor-dependent, the receptor was blocked with met-Rantes (#335-RM/CF, R&D Systems Minneapolis, MN, USA), a CCL5 analogy that has been shown to block the binding of CCL5 to its receptor CCR5 and inhibit leukocyte chemotaxis [40].

### 4.8. Flow Cytometry and Cell Sorting

Flow cytometry was performed using Canto II (BD Scientific Canto II Flow Cytometer), and data analysed with FlowJo (FlowJo, Version 9, Ashland). Cells were sorted on the Moflo Astrios EQ (Beckman Coulter Life Sciences MoFlo Astrios EQ Cell Sorter) by the FCCS (Flow Cytometry and Cell Sorting Facility) of the Department for BioMedical Research (Bern, Switzerland) prior to ICP-MS. Cell suspensions were stained in FACS buffer (1xPBS supplemented with sterile 2% FBS). The following monoclonal fluorochrome-conjugated antibodies were used for flow cytometry analysis: anti-CD45.2 (#109814, BioLegend, San Diego, CA, USA), anti-F4/80 (#123117, BioLegend San Diego, CA, USA), anti-CD64 (#139323, BioLegend San Diego, CA, USA), anti-Ly6C (#128005, BioLegend San Diego, CA, USA), anti-Ly6G (#127618, BioLegend San Diego, CA, USA), anti-CD11b (#553311, BD Biosciences, Franklin Lakes, NJ, USA), and Live/Dead Fixable Cell stain (#L34963, Thermo Fischer Scientific Waltham, MA, USA), anti-CD45.2 (#109819, BioLegend San Diego, CA, USA), anti-CD11b (#25-0112-81, Thermo Fisher Scientific Waltham, MA, USA), anti-F4/80 (#45-4801-80, Thermo Fisher Scientific, Waltham, MA, USA), anti-Ly6G (#551461, BD Biosciences Franklin Lakes, NJ, USA), anti-CD4 (#553653, BD Biosciences Franklin Lakes, NJ, USA), anti-Ly6C (#128005, BioLegend San Diego, CA, USA), anti-Ly6C (#45-5932-82, Thermo Fisher Scientific Waltham, MA, USA), anti-CD195 (#130-122-944, Miltenyi Biotec Auburn, CA, USA,), anti-CD45.1 (#553775, BD Biosciences Franklin Lakes, NJ, USA), anti-Ly6G (#17-9668-80, Thermo Fisher Scientific, Waltham, MA, USA), annexin V (#640920, BioLegend San Diego, CA, USA), and Fixable viability dye (eFluor^TM^506 CYAN, # 65-0866-14, eBioscience, San Diego, CA, USA). For liver tissue analysis, a total of 1 × 10^6^ cells were acquired for each sample. For blood samples, 4 × 10^5^ cells were acquired in each sample.

### 4.9. Statistics

All data are presented as mean ± SEM. Data distribution was assessed using the Kolmogorov–Smirnov test. Data were analysed using ordinary one-way ANOVA with Bonferroni’s multiple comparisons. At least three independent experiments in triplicate were performed for the in vitro studies. Statistical significance was accepted at *p* ≤ 0.05. Statistical significance is noted in figures by * *p* < 0.05, ** *p* < 0.01, *** *p* < 0.001, **** *p* < 0.0001. Analyses were performed using GraphPad PRISM 8.0 (PRISM, RRID:SCR_005375, Inc., La Jolla, CA, USA).

## Data Availability

The datasets generated during and/or analysed during the current study are available from the corresponding author on reasonable request.

## Supplementary Materials

The following supporting information can be downloaded at: https://www.mdpi.com/article/10.3390/ijms23052666/s1.
