# Supplementary material for: Kupffer Cells and Blood Monocytes Orchestrate the Clearance of Iron–Carbohydrate Nanoparticles from Serum
Source: Int J Mol Sci. 2022 Feb 28;23(5):2666. doi: 10.3390/ijms23052666 (PMC8910242; doi:10.3390/ijms23052666)
Supplement: Supplementary file 1 [file ijms-23-02666-s001.zip › ijms-1515219-supplementary.pdf]

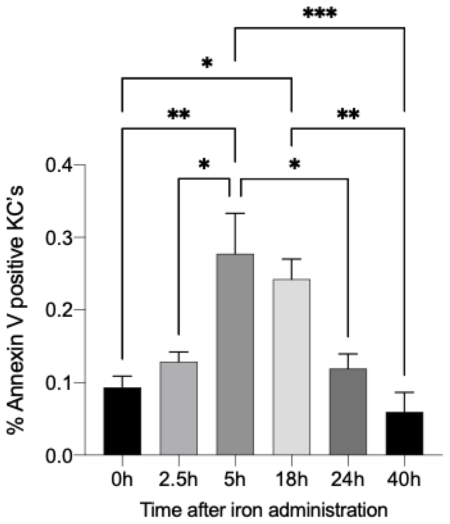

Figure S1. Time-dependent transient Annexin V expression in liver KCs.

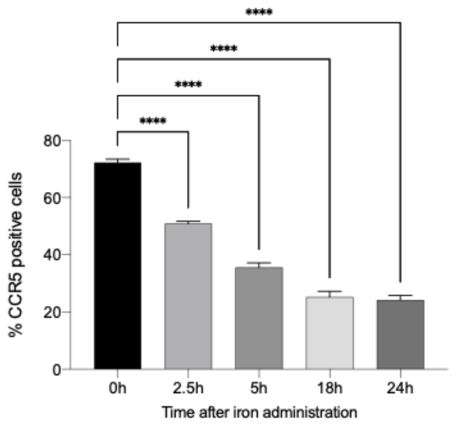

Figure S2. CCR5 expression in liver KCs.
